# siRNA–Mediated Methylation of *Arabidopsis* Telomeres

**DOI:** 10.1371/journal.pgen.1000986

**Published:** 2010-06-10

**Authors:** Jan Vrbsky, Svetlana Akimcheva, J. Matthew Watson, Thomas L. Turner, Lucia Daxinger, Boris Vyskot, Werner Aufsatz, Karel Riha

**Affiliations:** 1 Gregor Mendel Institute of Molecular Plant Biology, Austrian Academy of Sciences, Vienna, Austria; 2 Institute of Biophysics, Czech Academy of Sciences, Brno, Czech Republic; 3 Ecology, Evolution, and Marine Biology Department, University of California Santa Barbara, Santa Barbara, California, United States of America; National Institute of Genetics, Japan

## Abstract

Chromosome termini form a specialized type of heterochromatin that is important for chromosome stability. The recent discovery of telomeric RNA transcripts in yeast and vertebrates raised the question of whether RNA–based mechanisms are involved in the formation of telomeric heterochromatin. In this study, we performed detailed analysis of chromatin structure and RNA transcription at chromosome termini in *Arabidopsis*. *Arabidopsis* telomeres display features of intermediate heterochromatin that does not extensively spread to subtelomeric regions which encode transcriptionally active genes. We also found telomeric repeat–containing transcripts arising from telomeres and centromeric loci, a portion of which are processed into small interfering RNAs. These telomeric siRNAs contribute to the maintenance of telomeric chromatin through promoting methylation of asymmetric cytosines in telomeric (CCCTAAA)_n_ repeats. The formation of telomeric siRNAs and methylation of telomeres relies on the RNA–dependent DNA methylation pathway. The loss of telomeric DNA methylation in *rdr2* mutants is accompanied by only a modest effect on histone heterochromatic marks, indicating that maintenance of telomeric heterochromatin in *Arabidopsis* is reinforced by several independent mechanisms. In conclusion, this study provides evidence for an siRNA–directed mechanism of chromatin maintenance at telomeres in *Arabidopsis*.

## Author Summary

Telomeres are protein–DNA structures that protect the ends of eukaryotic chromosomes. A failure in this protective structure can lead to chromosomal instabilities and contribute to cancer and aging. The protective nature of telomeres relies on complex interactions between repetitive telomeric DNA and associated proteins. One major question is how telomeric proteins, including telomere-associated nucleosomes, are modified in order to achieve this protection. In this study, we have discovered that *Arabidopsis* telomeric nucleosomes contain a unique mixture of both active and inactive chromatin marks. Additionally, the telomeric DNA itself is modified by methylation of cytosines within the telomeric repeat. Regulation of DNA methylation is achieved by telomeric repeat–containing small RNAs, which are derived from the processing of telomeric transcripts by the RNA–dependent DNA methylation pathway. From these data, we infer that the formation of a proper telomere structure is partly regulated by non-coding telomeric RNAs.

## Introduction

Telomeres safeguard the stability of eukaryotic chromosomes by protecting natural chromosome ends from triggering DNA damage responses. Chromosome termini consist of telomeric and subtelomeric repeats that are bound by a specific set of telomere binding proteins as well as nucleosomes that exhibit features of pericentric heterochromatin [1]. These regions are usually devoid of functional genes, and transgenes integrated in the vicinity of telomeres are subjected to transcriptional silencing, a phenomenon known as telomere position effect [2]. Studies in mammals indicate that telomeric heterochromatin plays an important function in chromosome end protection and telomere length regulation. Inactivation of the SIRT6 histone deacetylase in human cells causes hyperacetylation of telomeric histone H3, telomere dysfunction and premature cell senescence [3]. Deficiency in histone methyltransferases or the retinoblastoma tumor suppressor leads to disruption of telomeric heterochromatin and aberrant telomere elongation in mouse cells [4]–[6]. Another important hallmark of heterochromatin in mammals is DNA methylation. Although vertebrate telomeric DNA does not appear to be methylated due to the lack of canonical CG sites, subtelomeric repeats are heavily methylated [7]. Interestingly, inactivation of DNA methyltransferases in mouse cells decreases 5-methylcytosine at subtelomeres and leads to increased telomeric recombination, without a concomitant change in histone modifications [7]. These data indicate a functional interaction between subtelomeric and telomeric chromatin.

Heterochromatin was thought to be transcriptionally inactive, but this view has been challenged by discoveries of numerous non-coding (nc) transcripts derived from heterochromatic loci. Some of these transcripts directly contribute to the assembly of heterochromatin at defined chromosomal domains and their biogenesis is vital for processes such as X chromosome inactivation, genomic imprinting, transposon silencing and centromere function [8]. Thus, it is not surprising that although telomeres possess marks of repressive heterochromatin, they are not transcriptionally silent. Recent studies revealed the presence of telomeric repeat-containing RNAs (TERRA) that are transcribed from subtelomeric regions in yeast and vertebrates [9]–[11]. TERRA are removed from telomeres either through Rat1p-dependent degradation in budding yeast or through non-sense mediated RNA decay (NMD) in human; deficiencies in these RNA processing pathways have dramatic effects on telomere maintenance [9], [10]. Hypomethylation of subtelomeric regions in mammalian cells lacking DNA methyltransferases leads to the overproduction of TERRA [11], [12]. This suggests that the epigenetic status of subtelomeres and telomeres influences TERRA expression.

The discovery of TERRA raised the question of whether ncRNAs contribute to the establishment of telomeric heterochromatin. This hypothesis gained support in a recent study in which downregulation of TERRA by exogenous short interfering RNAs (siRNAs) in human cell lines led to depletion of histone heterochromatic modification from telomeres [13]. In many organisms, RNA-mediated chromatin silencing relies on small RNA molecules that guide effector complexes to target sites [8], [14]. However, involvement of small RNAs in chromatin formation at canonical telomeres has not been shown yet. In this study, we investigate chromatin organization and transcription at chromosome ends in the model plant *Arabidopsis thaliana*. We detect the presence of transcripts containing telomeric repeats and show that some of these transcripts are processed into ∼24 nt siRNAs. These transcripts are produced from telomeres as well as from intrachromosomal telomeric loci that are mainly located at centromeres. The 24 nt siRNAs are generated through the RNA-dependent DNA methylation (RdDM) pathway, which is a plant-specific mechanism that utilizes siRNAs to guide DNA methyltransferases to asymmetric cytosines (CNN) [15], [16]. We demonstrate that RdDM is responsible for methylation of telomeric DNA that contains cytosines exclusively in asymmetric sequence contexts and hence for reinforcement of heterochromatic marks at telomeres.

## Results

### Chromatin organization at *Arabidopsis* chromosome termini

**Figure 1:**
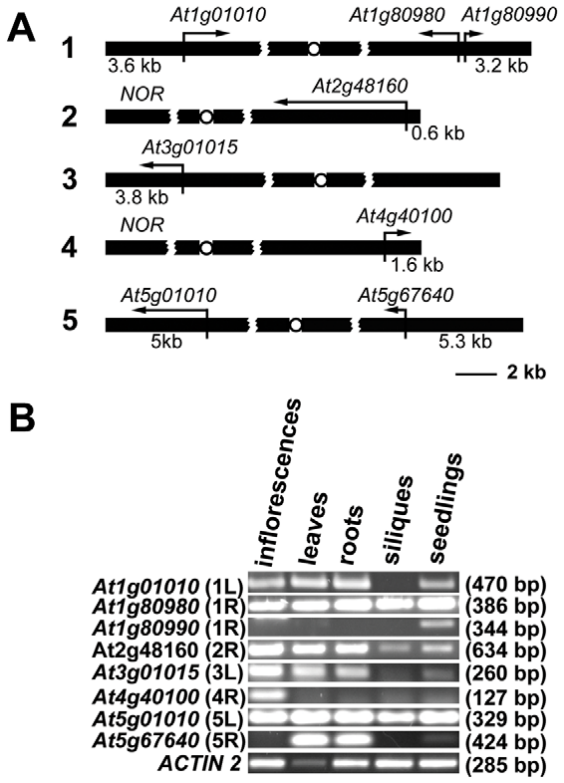
Expression of *Arabidopsis* chromosome-terminal genes. (A) A diagram of gene arrangement at the ends of five *Arabidopsis* chromosomes. Arrows illustrate the relative size and direction of transcripts of annotated terminal genes. The distance of the predicted ATG codon from the telomere is indicated. (B) Expression of the terminal genes in different tissues of wild-type plants assayed by RT-PCR. The size of the PCR products is indicated in parenthesis.

Gene organization at chromosome ends in *Arabidopsis* appears to be unique. In contrast to the majority of organisms with known telomere/subtelomere sequences, 8 of the 10 *Arabidopsis* subtelomeres have no repetitive DNA, and predicted genes are annotated in the immediate vicinity of telomeres [17] (Figure 1A). We experimentally confirmed that sequences annotated as chromosome ends are indeed associated with telomeres for 7 chromosome arms with the exception of the right arm of chromosome 3 [18]. The two remaining chromosome termini contain clusters of ribosomal RNA genes (NORs) [19]. We performed reverse transcription (RT) PCR analysis to verify that all the predicted terminal genes are expressed and that they do not represent pseudogenes (Figure 1B). The genes showed distinct tissue-specific expression patterns and the size of the RT-PCR products corresponded to the predicted size of the spliced mRNAs. There was no obvious correlation between the level of expression and promoter distance from telomeres, and even the *At2g48160* gene, with a promoter immediately adjacent to telomeric DNA, was robustly expressed. These data indicate that, in contrast to yeast and mammals, *Arabidopsis* telomeres do not silence genes located in their vicinity.

**Figure 2:**
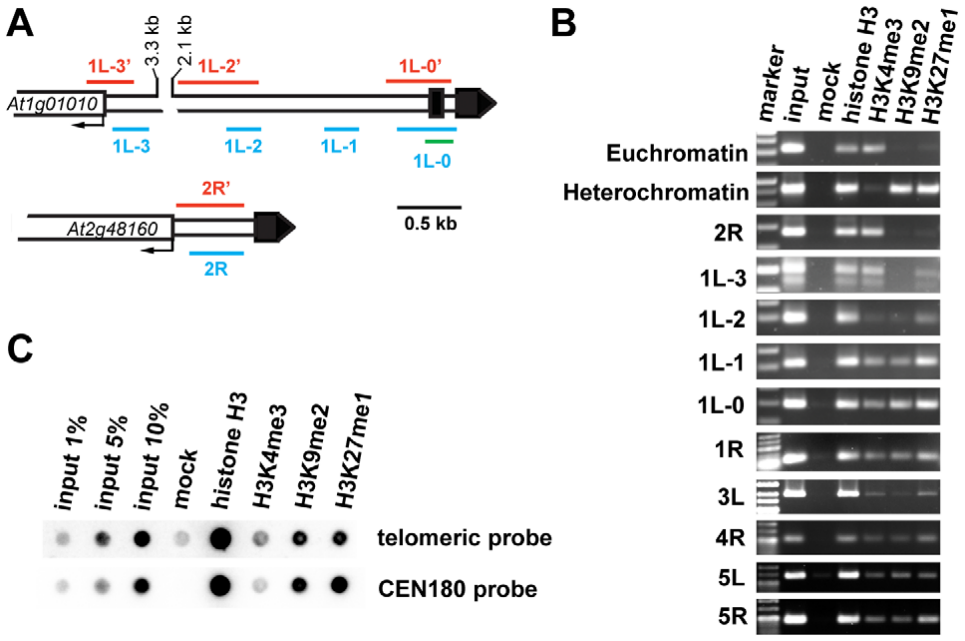
Chromatin structure of *Arabidopsis* chromosome termini. (A) Schematic diagram of the 1L and 2R chromosome ends. Black boxes represent telomeric DNA. The red and blue bars span regions analyzed by bisulfite sequencing and ChIP PCR, respectively. The green bar indicates the region analyzed by ChIP qPCR. (B) ChIP PCR data showing the distribution of methylated histones at unique regions immediately adjacent to telomeres at the indicated chromosome arms. A euchromatic fragment of the *MEE51* gene and a heterochromatic gypsy-like retrotransposon (At4g03770) were amplified from the ChIP fractions as a control. (C) Immunoprecipitated DNA analyzed by sequential dot-blot hybridization with a telomeric probe and the centromeric CEN180 probe.

The high transcriptional activity near telomeres raised questions about the chromatin structure of chromosome termini in *Arabidopsis*. We investigated the distribution of histone modification marks typical for plant euchromatin (tri-methylation of histone H3 at Lys4, H3K4me3) and heterochromatin (di-methylation of H3 at Lys9, H3K9me2; and mono-methylation of H3 at Lys27, H3K27me1) at telomere-associated regions by chromatin immunoprecipitation (ChIP). The ∼600 bp region immediately adjacent to the telomere on the right arm of chromosome 2 (2R) represents the promoter of the *At2g48160* gene (Figure 2A) and carries typical euchromatic histone marks (Figure 2B). The H3K4me3 euchromatin mark was also dominant at the promoter of the *At1g01010* gene that is located ∼3.5 kb from the telomere on the left arm of chromosome 1 (region 1L-3, Figure 2A and 2B), although we could detect a weak H3K27me1 signal that is usually typical of heterochromatin. Histone heterochromatic marks (H3K9me2 and H3K27me1) became more pronounced at the 1L-2 and 1L-1 regions that are located on the same arm ∼1.5 kb and 1 kb from the telomere, respectively (Figure 2A and 2B). The 1L telomere contains a recent 104 bp insertion of mitochondrial DNA embedded within the centromere-proximal region of telomeric repeats [20] (Figure 2A). Using this insertion to design primers that span the centromere-proximal part of the 1L telomere (1L-0, Figure 2A), we were able to demonstrate that this region also displays heterochromatin marks (Figure 2B). Nevertheless, the 1L-0 region still possessed clearly detectable H3K4me3, which is atypical of classical heterochromatin where the H3K4me3 modification is strongly reduced in comparison to H3K27me1 and H3K9me2. A similar histone-modification pattern was also observed in telomere-adjacent regions of five other chromosome arms (Figure 2B).

To further examine chromatin at telomeres, we analyzed ChIP fractions by dot-blot hybridization with a telomeric probe (Figure 2C). The *Arabidopsis* genome is enriched for intrachromosomal degenerated telomeric repeats that are mainly localized at centromeres (Figure S1). To specifically assay for chromatin at telomeres, we used stringent hybridization conditions at which the centromere-derived signal is eliminated to less than 2% of the total telomeric signal (Figure S1). We readily detected H3K27me1 and H3K9me2 modifications, and a weaker but still clearly detectable H3K4me3 signal. This hybridization pattern was reminiscent of the results obtained by ChIP analysis of telomere-adjacent regions by PCR (Figure 2B). Thus, our ChIP data show that *Arabidopsis* telomeres form chromatin that is enriched for H3K9me2 and H3K27me1 heterochromatic marks, but still retains the euchromatic H3K4me3 modification. We found that the heterochromatin marks extend ∼1.5 kb into the subtelomeric region of 1L. A survey of a high-resolution genome-wide map of H3K9me2 distribution indicates that H3K9me2 also spreads up to 1.5 kb from telomeres at chromosome arms 1R, 3L, 4R and 5L [21] (http://epigenomics.mcdb.ucla.edu/H3K9m2/). However, detecting the prominent H3K4me3 signal side by side with the heterochromatic marks (Figure 2B and 2C) strongly indicates that *Arabidopsis* telomeres exhibit features of intermediate heterochromatin that is characterized by retention of opposing histone H3 methylation marks [22].

### Identification of telomeric DNA–containing transcripts and siRNAs

**Figure 3:**
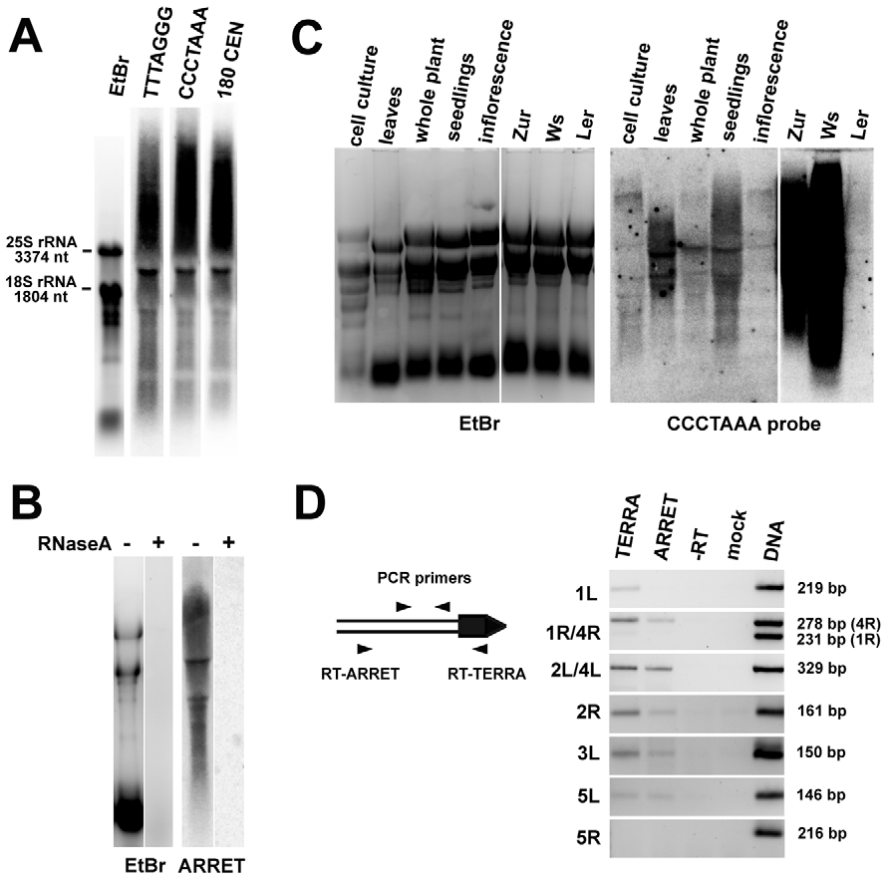
Identification of TERRA and ARRET transcripts in *Arabidopsis*. (A) Northern blot analysis of wild-type RNA that was hybridized with a strand-specific TTTAGGG probe, stripped after exposure further sequentially rehybridized with CCCTAAA and the centromeric CEN180 probe. The gel stained with ethidium bromide (EtBr) is shown as a loading control. (B) Sensitivity of ARRET transcripts to RNaseA. (C) Northern blot detection of TERRA in different tissues of wild-type Col plants and in seedlings of the Ws, Zur and Ler ecotypes. The left and right parts of the membrane were exposed for 1 day and 2 h, respectively. (D) Detection of telomere-derived TERRA and ARRET transcripts by RT-PCR. The diagram outlines the strategy used for strand-specific RT-PCR at a hypothetical chromosome end (the telomere is indicated as a black box). The size of the expected PCR product for each telomere is indicated. As chromosome ends 1R and 4R contain a stretch of sequence homology, one set of primers was used to assay for the expression at both telomeres in one reaction. The resulting chromosome-end-specific products can be distinguished by their size. It is currently unknown whether the subtelomere sequence at the NOR-bearing chromosome end represents the 2L or 4L telomere. ARRET transcripts at this arm were not analyzed because they correspond to the nascent 45S rRNA.

We next asked whether *Arabidopsis* telomeres are transcribed by assaying for the presence of TERRA by Northern hybridization with a CCCTAAA probe. We readily detected two types of TERRA: heterogeneous transcripts which ranged from high molecular weight strands that migrated at the limits of gel resolution to hundreds of nucleotides, and several distinct bands (Figure 3A). We also detected antisense telomeric transcripts (ARRET) that gave a similar hybridization pattern as the TERRA by the complementary TTTAGGG probe (Figure 3A). These signals disappeared after pretreatment of the samples with RNaseA (Figure 3B and data not shown) demonstrating that they do not represent remnants of DNA in RNA preparations. Expression of TERRA varied between RNA samples extracted from different tissues of *Arabidopsis* (Figure 3C). Interestingly, remarkable variation in expression was also detected between different *Arabidopsis* accessions, as the levels of TERRA in seedlings of Zur and Ws ecotypes were almost two orders of magnitude higher than in Col and Ler (Figure 3C). *Arabidopsis* TERRA and ARRET can originate at telomeres or arise from transcription of degenerated intrachromosomal telomeric sequences localized at centromeric regions (Figure S1). The bulk of centromeric DNA consists of 177–179 bp satellite repeats (CEN180), a subset of which is transcribed [23]. Sequential hybridization of a Northern blot with probes detecting TERRA and CEN180 resulted in an almost identical hybridization pattern, characterized by five distinct bands (Figure 3A). Hybridization of the blots with probes detecting sequences immediately adjacent to telomeres did not produce any detectable signal (data not shown). These results suggest that TERRA and ARRET transcripts detected by Northern analysis mainly arise from centromeric regions that contain remnants of telomeric DNA and not from the transcription of telomeres.

To examine whether telomeres are transcribed at levels non-detectable by Northern hybridization, we analyzed expression of subtelomeric regions adjacent to telomeric DNA by strand-specific RT-PCR in flowers. We could distinguish expression of TERRA and ARRET by using either telomeric or subtelomeric arm-specific primers for reverse transcription (Figure 3D). We detected expression of both TERRA and ARRET at four out of eight analyzed chromosome arms. We failed to detect any transcription at chromosome arms 1R and 5R. Interestingly, only the TERRA but not ARRET transcripts were detected at 1L. The RT-PCR data demonstrate that at least five *Arabidopsis* telomeres are indeed transcribed, albeit at a low level. To gain further insights into telomere transcription, we cloned a ∼500 nt promoter of the *At2g48160* gene, which is located next to the telomere (Figure 1), in front of a reporter β-glucuronidase (*GUS*) gene in both sense and antisense orientations. We could detect GUS transcripts in transgenic plants carrying both constructs, although the expression in the antisense direction was much weaker than in the sense orientation (Figure S2). This experiment further supports the idea that telomere adjacent regions can drive transcription into a telomere.

**Figure 4:**
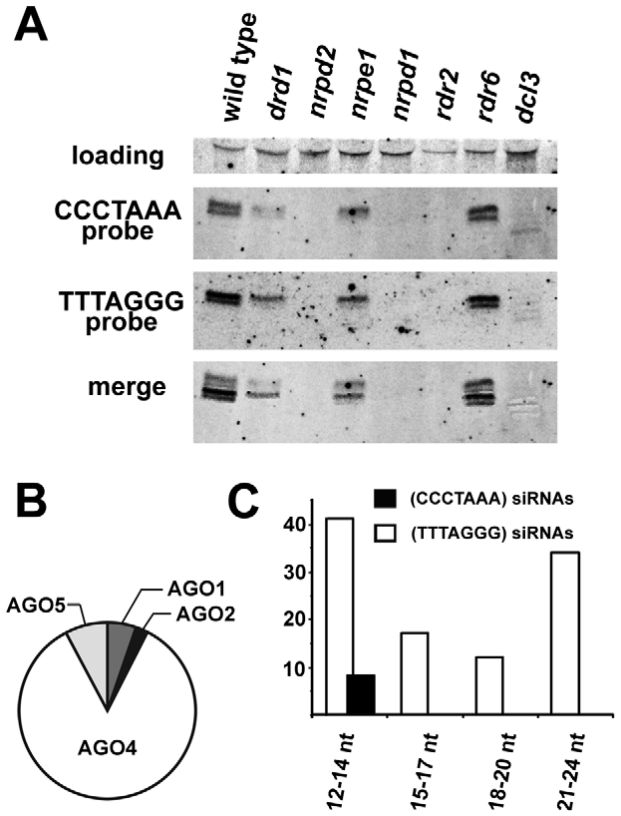
Detection of telomeric siRNAs. (A) Northern analysis of small RNAs from wild-type and the indicated RdDM mutants. The membrane was hybridized with a CCCTAAA probe, stripped and rehybridized with the TTTAGGG probe. Electronically merged autoradiograms show faster migration of the C-siRNA that is due to a sequence bias towards pyrimidines. The loading control represents a large RNA that hybridizes to the TTTAGGG probe. (B) Distribution of siRNAs containing at least 12 nt of telomeric sequence in different Argonaute complexes. (C) Distribution of AGO4-associated C- and G-siRNAs according to the extent of homology to telomeric sequence. The total number of siRNAs is indicated on the y-axis.

The presence of centromeric and telomeric TERRA and ARRET indicated that telomeric transcripts are able to form partially double stranded (ds) intermediates that could be processed by a Dicer into siRNA. In support of this hypothesis, siRNAs corresponding to both strands of telomeric DNA were detected in wild-type plants (Figure 4A). We estimate the size of the telomeric C-rich strand siRNAs (C-siRNA) to be 24–25 nt, and the size of G-siRNAs to be 23–24 nt (Figure S3). The formation of 24 nt siRNAs in *Arabidopsis* is mediated by RNA-processing enzymes of the RdDM pathway [24]. This pathway is specific to plants and mediates methylation of cytosine residues in an asymmetric sequence context (CNN). The absence of telomeric 23–25 siRNAs in plants lacking RNA-dependent RNA polymerase 2 (RDR2), Dicer-like 3 (DCL3) or subunits of RNA Polymerase IV (NRPD1 or NRPD2) and their reduction in two other RdDM mutants (*drd1* and *nrpe1*) further demonstrated that telomeric siRNAs belong to the category of 24 nt heterochromatic siRNAs (Figure 4A). These siRNAs are usually derived from heterochromatic loci and form the most abundant fraction of plant small RNAs [25], [26]. They typically associate with Argonaute 4 (AGO4) that is part of the effector complex that, together with Polymerase V, mediates CNN methylation [27], [28]. To determine whether telomeric siRNAs associate with AGO4, we surveyed published datasets containing ∼600,000 Argonaute (AGO1, AGO2, AGO4 and AGO5)-bound small RNAs [29]. We identified a total of 133 small RNAs containing at least 12 nucleotides with a perfect telomeric repeat (Table S1). As expected, the majority of these small RNAs were associated with AGO4 (Figure 4B). Surprisingly, the AGO4-associated telomeric siRNAs were almost exclusively G-siRNAs and only a few C-siRNAs containing no more than 14 nt of the CCCTAAA repeat sequence were found in the dataset (Figure 4C). Since the levels of total G- and C-siRNAs are similar (Figure 4A), this bias may be caused by a selective incorporation of the G-siRNAs into the AGO4 complex.

**Figure 5:**
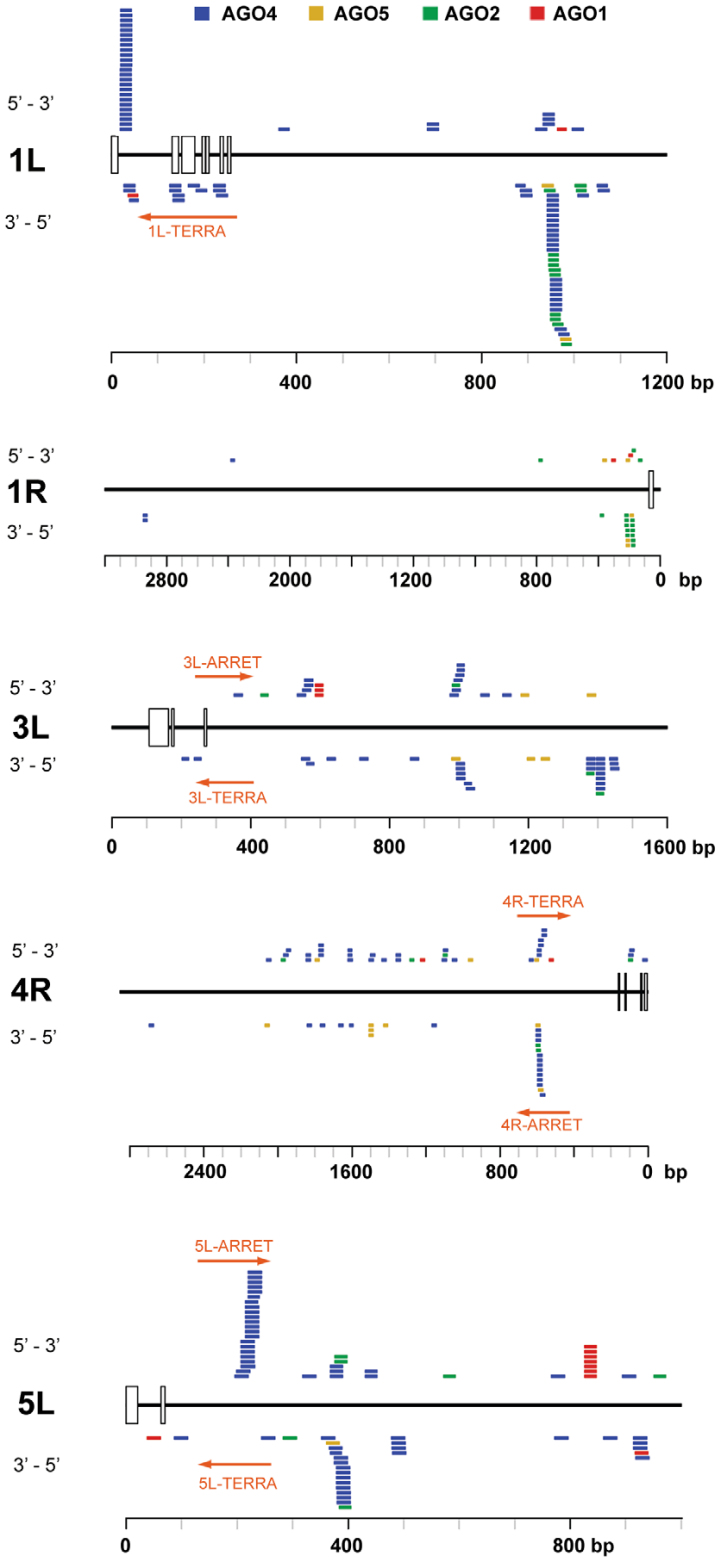
Distribution of Argonaute-associated siRNAs in telomere-adjacent regions. The diagrams represent subtelomeric regions at the indicated chromosome arms. The rulers indicate the distance from a continuous array of telomeric repeats, open boxes mark positions of telomeric repeats intermingled within subtelomeric sequences. Only chromosome arms containing siRNAs detected within 5 kb from telomeres are shown. The position and orientation of AGO-associated siRNAs is indicated by colored bars. Only siRNAs that aligned to unique locations are included (Table S2). The TERRA and ARRET transcripts detected by strand-specific RT-PCR are depicted by a red arrow.

As TERRA transcripts are produced from telomeres as well as from centromere-located telomeric DNA, the siRNAs may be of either telomeric or centromeric origin. To determine whether telomere-derived transcripts are processed into siRNAs, we aligned Argonaute-associated siRNAs with telomere-adjacent sequences. We found abundant siRNAs corresponding to both strands of subtelomeric DNA at chromosome arms 1L, 1R, 3L, 4R and 5L (Figure 5, Table S2). Since these regions are formed by unique sequences, the origin of the siRNAs can be unambiguously traced to these loci. Interestingly, AGO4-associated siRNAs were particularly enriched at the chromosome ends that also exhibited expression of TERRA and ARRET (1L, 3L, 4R, 5L; Figure 5). These data strongly argue that telomeric TERRA and/or ARRET are processed into siRNAs.

### The RdDM pathway mediates methylation of telomeric DNA

**Figure 6:**
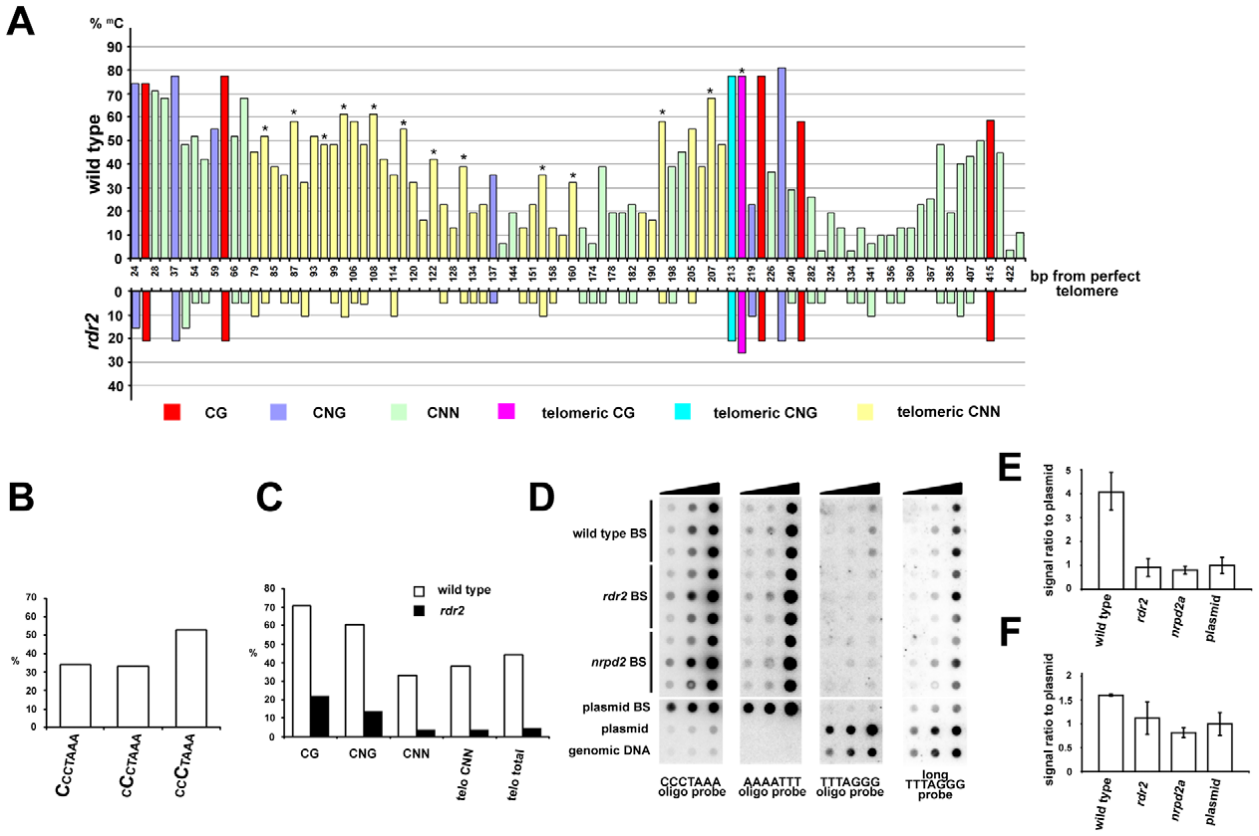
Methylation of telomeric DNA in wild-type and RdDM-deficient plants. (A) The chart shows the frequency and distribution of cytosine methylation in the 1L-0' region. In total, 30 clones from five independently treated wild-type samples and 19 clones from three independent *rdr2* samples were analyzed. Asterisks indicate the third cytosine in the CCCTAAA sequence. (B) The proportion of methylated cytosines in the 1L-0' region of wild-type plants depending on the position within the telomeric repeat as determined by bisulfite sequencing. (C) Frequency of cytosine methylation in the whole 1L-0' region according to sequence context in wild-type and *rdr2* plants. (D) Cytosine methylation in bulk telomeric DNA assayed by dot-blot hybridization. Bisulfite-treated (BS) genomic DNA was spotted onto a membrane (∼7, 33 and 200 ng from each sample) and sequentially hybridized with AAAATTT, TTTAGGG and CCCTAAA probes. Untreated wild-type genomic DNA, and untreated and bisulfite-treated (BS) plasmids carrying ∼750 nt of *Arabidopsis* telomeric DNA were used as controls. (E,F) Quantification of signals obtained with oligo (E) and long (F) TTTAGGG probes. The signal intensity of non-converted DNA (obtained with the TTTAGGG probe) was normalized to the amount of telomeric DNA determined from hybridization with the CCCTAAA probe. The signal from BS-treated plasmid served to determine background hybridization of the probes to fully converted non-methylated telomeric DNA. Error bars represent standard deviation (N = 3).

Plants can methylate cytosines in all sequence contexts, and DNA methylation at asymmetric positions relies largely on 24 nt siRNAs and on the RdDM pathway. The presence of telomeric siRNAs prompted us to ask whether telomeric DNA, which contains cytosines exclusively in the CNN context, can be methylated. We took advantage of the unique insertion in the 1L telomere that allowed us to design primers spanning 13 CCCTAAA repeats located in the centromere-proximal part of the 1L telomere (region 1L-0'; Figure 2A). Bisulfite sequencing of the 1L-0' region in wild-type plants revealed that over 40% of cytosines in these telomeric repeats are methylated (Figure 6). In contrast, the 1L and 2R subtelomeric regions are devoid of DNA methylation (Figure S4). The telomeric methylation in 1L-0' is non-randomly distributed, with preferential enrichment at the third cytosine in the CCCTAAA sequence (Figure 6A and 6B). A similar observation was recently made through whole genome bisulfite sequencing that also revealed methylation of telomeric repeats, albeit at a lower total frequency than reported here [30]. The level of 5-methylcytosine in all sequence contexts was dramatically reduced in *rdr2* mutants, arguing that methylation of the 1L-0' region primarily depends on the RdDM mechanism (Figure 6A and 6C).

We next examined whether cytosine methylation and its dependence on the RdDM pathway is a general feature of telomeric DNA. We sequentially hybridized bisulfite-treated total genomic DNA to oligonucleotide probes that first detected fully converted telomeric DNA (probe AAAATTT), then unconverted, and thus completely methylated DNA (probe TTTAGGG), and finally the complementary cytosine-free strand (probe CCCTAAA) as a control for loading (Figure 6D). A strong hybridization AAATTTT signal suggested that the bulk of telomeric DNA is only weakly methylated. Nevertheless, a portion of wild-type DNA was resistant to bisulfite conversion as hybridization with the TTTAGGG oligo probe showed a signal that was ∼4-fold higher than a background signal from a corresponding amount of non-methylated bisulfite-converted telomeric DNA cloned in a plasmid (Figure 6D and 6E). These data further indicate the presence of some heavily methylated CCCTAAA sequences in wild-type plants. Importantly, this CCCTAAA signal was reduced to a background level in *rdr2* and *nrpd2a* mutants (Figure 6D and 6E). To further investigate whether methylation occurs at telomeres, we performed high-stringency hybridization of the bisulfite-converted samples with a long telomeric TTTAGGG probe (Figure 6C). Under these conditions, converted plasmid-cloned telomeric DNA produces a high background hybridization signal that is likely caused by sufficiently stable interactions between longer fragments of the (TTTTAAA)_n_ converted telomeric DNA and the (TTTAGGG)_n_ probe. Nevertheless, wild-type DNA samples still produced a signal that was significantly higher than the background hybridization (Figure 6F). These data, together with the bisulfite sequencing of the 1L-0' telomeric region, strongly argue that DNA methylation is a general characteristic of *Arabidopsis* telomeres and that its maintenance requires the RdDM pathway.

**Figure 7:**
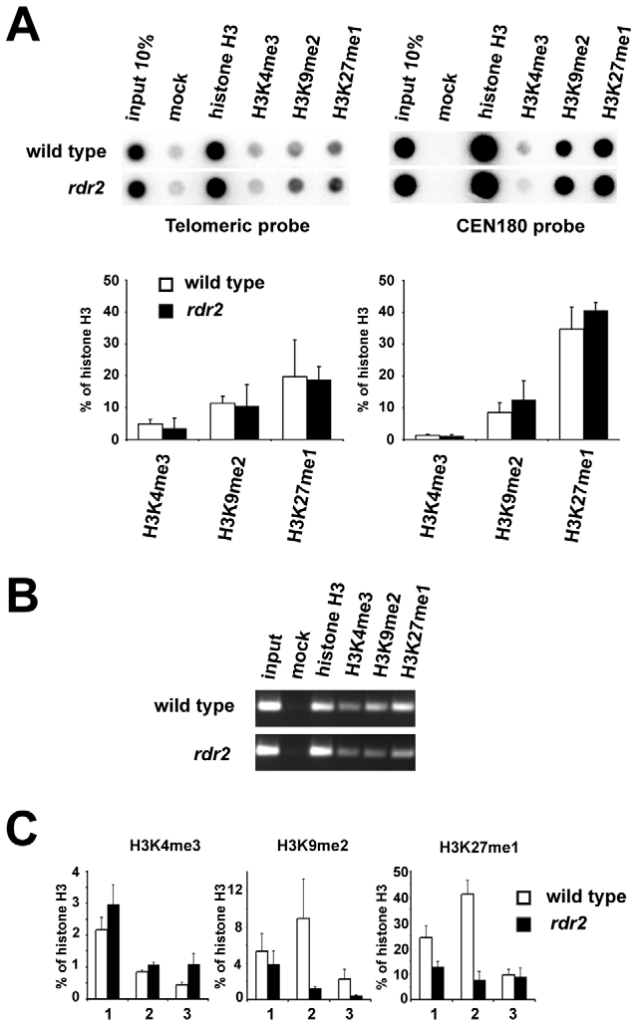
Telomeric heterochromatin in RdDM mutants. (A) Representative ChIP data of wild-type and *rdr2* plants. Chromatin was immunoprecipitated with antibodies against histone H3, H3K4me3, H3K9me2 and H3K27me1, blotted onto a membrane and hybridized with the TTTAGGG probe. The same membrane was stripped after exposure and rehybridized with the centromeric CEN180 probe. Data from three independent ChIP experiments were used for quantification. Signals were normalized to mock. (B) Analysis of ChIP fractions from wild-type and *rdr2* plants by PCR with primers spanning the 1L-0 locus. (C) qPCR analysis of H3K4me3, H3K9me2 and H3K27me1 at the 1L-0 locus in wild-type and *rdr2* plants. Each value represents an average of three qPCR measurements normalized to histone H3 occupancy for each ChIP fraction. The results of three independent pairwise ChIP experiments (1, 2 and 3) are presented.

Loss of DNA methylation is often accompanied by chromatin remodeling. However, the decrease in telomeric DNA methylation did not result in a significant loss of heterochromatic histone marks, and both H3K9me2 and H3K27me1 remained enriched at the bulk of telomeric DNA in *rdr2* mutants (Figure 7A). However, analysis of histone modifications at the 1L-0' locus by ChIP and quantitative PCR (Figure 7B and 7C) showed a decrease in H3K9me2 and H3K27me1 (Figure 7C) in *rdr2* mutants. These data indicate that although the RdDM-dependent mechanism is not solely responsible for heterochromatin formation at telomeres, it contributes to its maintenance by mediating methylation of telomeric DNA, thereby reinforcing heterochromatic histone modifications.

Disruption of telomeric heterochromatin or demethylation of subtelomeric sequences leads to increased telomere elongation and recombination in mouse [7]. Our analysis of telomere length and intrachromatid recombination at chromosome ends did not reveal any differences between RdDM mutants and wild-type plants (Figure S5 and Figure S6). This observation further corroborates our finding that despite reduced DNA methylation, the bulk of telomeric chromatin in *rdr2* mutants still retains heterochromatic features.

## Discussion

Heterochromatin is a universal characteristic of chromosome termini in a variety of organisms, including yeast, flies and mammals. Subtelomeric regions in these organisms are gene-poor and enriched for middle to highly repetitive sequences that contribute to the formation of a heritably repressed chromatin structure at chromosome termini that shares similarities with pericentromeric heterochromatin [1], [31], [32]. Nevertheless, some aspects of chromatin organization appear to be unique at telomeres as telomeric chromatin in humans and plants display unusually short nucleosomal spacing (∼160 nt) in comparison with the ∼180 nt periodicity at the bulk of chromatin [33]–[35].

In contrast to many other organisms, telomeres in *Arabidopsis* are directly adjacent to transcriptionally active genes. This situation is more similar to silenced transposons inserted in gene-rich regions than to pericentromeric heterochromatin. This is also reflected in the organization of telomeric chromatin that exhibits features of intermediate heterochromatin that is characterized by the presence of both active and repressive histone H3 marks. Such chromatin was described to be associated with some *Arabidopsis* transposons and transgenic loci [22], [36]. Chromatin analysis of the 1L subtelomere demonstrates that repressive histone H3 modifications are most pronounced immediately next to telomeres and that their presence gradually recedes with growing distance from telomeres. Data on whole-genome distribution of H3K9me2 indicate that this also holds true for telomere-associated regions of several other chromosome arms [21]. These data infer that repressive histone marks are primarily established at telomeres and spread only a limited distance within adjacent subtelomeric sequences. The existence of such relatively small clusters of repressive chromatin (2–5 kb) next to otherwise large gene-rich regions suggests a functional importance for the heterochromatization of telomeres in *Arabidopsis*. It further suggests the existence of mechanisms that specifically maintain repressive histone modifications at telomeres. Assembly of heterochromatin at chromosome ends in budding yeast is partially dependent on tethering Sir proteins to telomeres via the Rap1 telomere-binding protein [31]. Human SIRT6 histone deacetylase preferentially associates with telomeres, although how it is recruited to chromosome termini is not known [3]. A recent study in mice overexpressing TRF2 indicates that, similar to the situation in yeast, heterochromatin formation at telomeres in mammals may also involve telomere-binding proteins [37].

The discovery of TERRA provides another attractive model that involves targeting the chromatin remodeling machinery to chromosome termini through ncRNA [38], [39]. This suggestion was recently corroborated by the finding that downregulation of TERRA by RNAi in human cells causes a decrease in histone H3K9 methylation [13]. It was proposed that TERRA facilitates heterochromatin formation by stabilizing interactions between heterochromatin factors and telomeric DNA. In this study, we demonstrate expression of telomeric transcripts in *Arabidopsis* and describe a mechanism by which telomeric repeats-containing RNAs affect telomeric chromatin through siRNA.

In contrast to the situation in mammals, where only UUAGGG telomeric transcripts were detected [10], [11], both telomeric strands appear to be transcribed from some telomeres in *Arabidopsis*. This indicates that canonical telomeric DNA may, under certain circumstances, act as a promoter and initiate transcription. Two lines of observations further corroborate the link between transcription and telomeric DNA in *Arabidopsis*. Firstly, short stretches of a telomeric sequence were found in numerous *Arabidopsis* promoters and it has been shown that these interstitial telomere motifs are required for transcription [40]. Secondly, several transcription factors have been identified in *Arabidopsis* that specifically bind to telomeric DNA in electromobility shift assays (reviewed in [41]). Thus, it is possible that some of these transcription factors localize to telomeres and promote their expression.

In addition to transcripts that originated at telomeres, we detected TERRA and ARRET that are apparently generated by transcription of centromere-associated telomeric loci. We cannot currently determine the exact identity of telomere- or centromere-derived TERRA/ARRET that is processed by DCL3 and degraded to telomeric siRNAs. The requirement of RDR2 for siRNA formation indicates that the predicted dsRNA intermediate is not a simple annealing product of complementary TERRA and ARRET, but is dependent on additional RNA-dependent RNA synthesis. Thus, even relatively low level transcripts can yield significant amounts of siRNA. In fact, direct detection of precursor transcripts in the RdDM pathway has been so far reported only in a special transgene system [42]. In plants, heterochromatic siRNAs serve to guide DNA methylases to specific asymmetric CNN positions in a mechanism that relies on AGO4 [28]. Interestingly, AGO4 appears to retain telomeric G-siRNAs, and not the complementary C-siRNAs, although these data should still be verified by Northern analysis of AGO4 co-immunoprecipitated siRNAs. It is unknown whether the bias towards G-siRNAs is of biological significance, but it is interesting that the AGO4 complex appears to specifically retain siRNAs complementary to the telomeric strand to be methylated.

Our data, showing methylation of bulk telomeric DNA as well as heavy methylation of the centromere-proximal region of the 1L telomere, together with data from whole genome bisulfite sequencing [30], argue that telomeric heterochromatin in *Arabidopsis* is not only defined by histone modifications, but also by DNA methylation. Although mammalian telomeres lack CG sites, and are, thus, believed to be unmethylated, at least two proteins linked to DNA methylation (SMCHD1, MBD3) have been found in purified fractions of human telomeric chromatin [43]. Additionally, the recent discovery of CNN and CNG methylation in human embryonic stem cells warrants the re-examination of DNA methylation at human telomeres [44].

We demonstrate that the maintenance of telomeric DNA methylation depends, to a large extent, on heterochromatic siRNA and the RdDM machinery. Intriguingly, loss of telomeric DNA methylation only has a slight effect on histone methylation at bulk telomeres, indicating that assembly of *Arabidopsis* telomeric heterochromatin relies on several reinforcing mechanisms that recruit histone methyltransferases such as SUVH4 to telomeres [45]. Loss of DNA methylation has a more profound effect on histone methylation at the centromere-proximal part of the 1L telomere. This indicates that RdDM may play a role in maintaining heterochromatin at the boundary between telomeres and adjacent euchromatic genes. The involvement of siRNA in modulation of telomeric heterochromatin may not be restricted to plants. Our data in *Arabidopsis* are reminiscent of the situation in fission yeast where heterochromatin in subtelomeric regions is established by two independent pathways, one of which relies on the telomere-binding protein Taz1, while the other involves RNA-induced transcriptional silencing (RITS) [46]. However, in contrast to the situation in *Arabidopsis* where siRNA targets canonical telomeric repeats, RITS in fission yeast is directed at centromere-like sequences that are located ∼15 kb from telomeres. In humans, TERRA has been proposed to act as a scaffold, reinforcing interactions between telomere-binding proteins and heterochromatin factors such as ORC1 and HP 1 [13]. Nevertheless, human TERRA could also promote heterochromatin formation through an siRNA-mediated pathway. This notion is supported by the observation that enrichment of Argonaute-1 at human telomeres is correlated with increased H3K9 methylation and HP1 association [47], and by the discovery of telomere-derived human siRNAs [48].

## Materials and Methods

### Plant material and growth conditions

*Arabidopsis* mutants carrying the following alleles were used in this study: *dcl3-1* (*dcl3*), *rdr2-1* (*rdr2*), *nrpd1a-4* (*nrpd1*), *nrpd1b-1* (*nrpe1*), *sgs2-1* (*rdr6*), *drd1-1* (*drd1*) and *nrpd2a-1* (*nrpd2*). Plants were grown in soil under long-day conditions (16 h light/8 h dark) at 22°C.

### RNA analyses

Total RNA was extracted using TriReagent solution (Sigma). For Northern blot analysis, 10 µg aliquots were separated on 1.2% formaldehyde agarose gels, blotted onto a nylon membrane and hybridized with [^32^P] 5′ end-labeled (TTTAGGG)_4_ (TTTAGGG probe) or (TAAACCC)_4_ (CCCTAAA probe) oligonucleotides. Oligo hybridizations were carried out at 55°C as previously described [49]. Centromeric transcripts were detected by hybridization with a [^32^P]-labeled CEN180 repeat unit amplified from *Arabidopsis* genomic DNA using primers CEN1 and CEN2 (Table S3). For RT-PCR analyses, ∼2 µg of total RNA was reverse transcribed by using oligo dT for gene expression. The (CCCTAAA)_3_ oligo or subtelomere-specific primers (Table S3) were used for RT of TERRA and ARRET, respectively. The respective cDNAs were amplified by 25–35 cycles of PCR with specific primers (Table S3). Small RNAs were isolated from inflorescences using the mirVana miRNA isolation kit (Ambion), separated on 15% polyacrylamide gels and electroblotted onto a nylon membrane. Telomeric siRNAs were detected by hybridization with either (TTTAGGG)_4_ or (TAAACCC)_4_ oligo probes in ULTRAhyb-Oligo hybridization buffer (Ambion) at 42°C. The artificial 25 and 23 nt siRNAs were synthesized by *in vitro* transcription using T7 RNA polymerase (MBI). The T7-TOP oligonucleotide (10 µM) was annealed to a template oligonucleotide (10 µM) as indicated in Figure S3. *In vitro* transcription was carried out with 30U of T7 RNA polymerase (MBI) and the annealed oligos (0.5 µM) in 50 µL of 1× Transcription buffer (MBI) supplemented with NTPs (10 mM) and RiboLock RNase inhibitors (MBI) for 60 min at 37°C. 25 µL of the reaction was separated on a 15% polyacrylamide gel, electroblotted onto a nylon membrane and analyzed by Southern hybridization.

### Analysis of DNA methylation

Genomic DNA was extracted from 4 week old plants with the DNAeasy Plant Maxi Kit (Qiagen). Bisulfite modification was performed using the EpiTect Bisulphite Kit (Qiagen) according to the manufacturer's instructions. The completeness of the conversion was tested by PCR amplification of a non-methylated genomic region [50]. Modified DNA was used as a template for PCR amplification with the primers indicated in Table S3. The PCR products were cloned into the pCR2.1 TOPO cloning vector (Invitrogen) and sequenced using a BigDye terminator and an ABI310 sequencer (Applied Biosystems). The sequence of the clones was analyzed with the software CyMATE [50]. The efficiency of cytosine conversion in the 1L-0' region in these samples was further controlled by either spiking genomic DNA with a bacterial plasmid containing a region that partially overlaps with 1L-0' or by sequence analysis of other genomic loci that are devoid of 5-methylcytosines. For methylation analysis at bulk telomeric DNA, bisulfite-modified genomic DNA was transferred onto a nylon membrane by vacuum-blotting. As a control, a bisulfite-modified plasmid containing 750 bp of plant non-methylated telomeric DNA was blotted onto the membrane in an amount that roughly corresponded to the total amount of telomeric DNA present in genomic samples (1 ng of the plasmid contained telomeric DNA equivalent to ∼260 ng of genomic DNA). The membrane was hybridized with the [^32^P] 5′ end-labeled (TTTAAAA)_4_ oligo (AAAATTT probe) in a standard hybridization buffer [49] at 40°C. The membrane was washed twice for 10 min at 40°C in 2× SSC followed by a 40 min wash in 1× SSC at 40°C. The membrane was exposed to a Kodak Phosphor screen (Biorad) and scanned with Molecular Imager FX (Biorad). The membrane was then stripped and sequentially rehybridized with the TTTAGGG and CCCTAAA oligo probes at 55°C as described [49]. The final rehybridization was performed at 65°C with a strand-specific (TTTAGGG)_n_ probe that was obtained by labeling of a 750 bp fragment of telomeric DNA with α-[^32^P]-GTP. The signals were quantified using QuantityOne software (Biorad).

### Chromatin immunoprecipitation

Chromatin isolation and immunoprecipitation were performed as described [51] using antibodies against histone H3 (Abcam; cat. no. ab1791), H3K9me2 (Abcam; cat. no. ab1220), H3K4me3 (Abcam; cat. no. ab8580) and H3K27me1 (provided by Thomas Jenuwein). The DNA was column-purified from immunoprecipitated chromatin and concentrated in 50 µl of elution buffer. For dot-blot analysis, 40 µl of the DNA was blotted onto a nylon membrane and analyzed by hybridization with a [^32^P]-labeled 750 bp (TTTAGGG)_n_ probe. For PCR analysis, 1 µl of the eluted DNA was amplified by 30 cycles of PCR with the primers specified in Table S1. Quantitative PCR analysis of the 1L-0 region was performed using the iQ5 Real Time PCR detection system (Biorad) and a 2× SensiMix Plus SyBR Kit (PeqLab).

### Analysis of Argonaute-associated siRNAs

The sequences of Argonaute-associated siRNAs were retrieved from the NCBI (accession number GSE10036). The individual AGO datasets were searched for the presence of siRNAs containing a string of at least 12 nucleotides of *Arabidopsis* telomeric repeats of any possible permutation. Telomeric siRNAs were copied into an Excel table and manually annotated. Subtelomeric siRNAs were identified by attempting to align all Argonaute-associated siRNAs to an ∼15 kb sequence from the ends of each Arabidopsis chromosome using the publicly available program SOAP [52]. The subtelomeric sequences were derived from the sequences of whole chromosomes available in TAIR, and from cloned fragments of telomere-associated sequences deposited in the Gene Bank (AB033278 and AM177017). Perfectly matching siRNA alignments were retained, and plotted using R.

### Cytology

Mitotic chromosomes prepared from pistils of wild-type plants were subjected to fluorescence in situ hybridization (FISH) with a Cy3-conjugated (CCCTAAA)_2_ PNA probe (Metabion) as previously described [53]. Chromosomes were examined using a Zeiss Axioscope fluorescence microscope equipped with a CCD camera.

### Telomere analyses

The PETRA assay was carried out with genomic DNA extracted from a fifth generation *tert* mutant plant [54] according to the published protocol [18]. Terminal restriction fragment analysis was performed as described [49], [55]. Analysis of intrachromatid telomeric recombination was performed by the t-circle amplification assay [56]. DNA extracted from *Arabidopsis ku70* [57] mutants was used as a positive control.

## Supporting Information

Figure S1 Localization of telomeric DNA in *Arabidopsis* centromeres. A survey of the *Arabidopsis* genome led to the identification of several regions carrying short stretches of telomeric sequence in the proximity of centromeres (Uchida et al., 2002; Vannier et al., 2009). Fluorescent *in situ* hybridization (FISH) on pachytene chromosomes further showed co-localization of CEN180 and telomeric signals at the centromere of chromosome 1 (Armstrong et al., 2002) (A) This centromere-localized telomeric DNA is also readily detectable by FISH on mitotic metaphase chromosomes. The picture shows a diploid metaphase figure with ten *Arabidopsis* chromosomes counterstained with DAPI (red). Green signals represent telomeric DNA; the chromosome pair carrying centromere-localized telomeric DNA is indicated by arrows. (B) The poor annotation of Arabidopsis centromeres precluded in silico identification of genomic loci carrying telomeric and CEN180 sequences in close proximity. To look for the presence of such loci in the Arabidopsis genome, we performed PCR with combinations of primers that flank the centromeric CEN180 satellite repeat (CEN1, CEN2) as well as with primers that anneal either to the C-rich or G-rich telomeric strands (TelC, TelG). The reaction with both centromeric primers resulted in a ladder whose periodicity corresponds to the size of the CEN180 repeat unit (180 bp; lane 1). Importantly, products were also amplified in reactions in which the CEN1 primer was combined with either of the telomeric primers (lanes 5 and 6), demonstrating that telomeric sequences are adjacent to the CEN180 repeat. Furthermore, strong amplification products were obtained in reactions containing a single telomeric primer (lanes 8 and 9), indicating the existence of sequences that contain telomeric repeats in inverted orientation. (C) Intrachromosomal telomeric sequences do not efficiently hybridize to a telomeric probe under the high stringency conditions. Genomic DNA was digested with TruI1 restriction endonuclease, blotted onto a membrane and hybridized to a TTTAGGG probe at high stringency conditions (65 °C). The membrane was stripped after exposure and rehybridized to an oligo TTTAGGG probe under low stringency conditions (55 °C). While under the low stringency of hybridization interstitial telomeric DNA (<0.5 kb) contributed to ∼21% of the total telomeric signal, these sequences were barely detectable when the membrane was rehybridized at high stringency and more than 98% of the signal was derived from terminal restriction fragments ranging between 2–4 kb. [Uchida W, Matsunaga S, Sugiyama R, Shibata F, Kazama Y, et al. (2002) Distribution of interstitial telomere-like repeats and their adjacent sequences in a dioecious plant, Silene latifolia. Chromosoma 111: 313–320. Vannier JB, Depeiges A, White C, Gallego ME (2009) ERCC1/XPF protects short telomeres from homologous recombination in Arabidopsis thaliana. PLoS Genet 5: e1000380. Armstrong SJ, Caryl AP, Jones GH, Franklin FC (2002) Asy1, a protein required for meiotic chromosome synapsis, localizes to axis-associated chromatin in Arabidopsis and Brassica. J Cell Sci 115: 3645–3655.]
(9.55 MB TIF)

Figure S2 Analysis of bidirectional transcription of the GUS reporter gene from the 2Rp promoter. (A) Schematic representation of constructs used for this experiment. A ∼500 bp genomic fragment (indicated by arrow) localized between the telomere and *At2g48160* was cloned in sense and antisense orientations in front of the GUS reporter gene containing a ∼200 nt intron (the intron is indicated by thin line; empty boxes represent exons). Resulting constructs (*2Rp:GUS* and *R2p:GUS*, respectively) were randomly inserted in *Arabidopsis* genome using Agorbacterium mediated transformation. T2 transgenic plants were analyzed for GUS expression by histochemical GUS assay and RT-PCR. Primers used for RT-PCR are indicated by arrows. (B) In total, T2 seedlings of 12 independent transgenic lines carrying the *2Rp:GUS* construct, and 11 lines with the *R2p:GUS* were analyzed by histochemical GUS assay for the presence of GUS enzymatic activity. Two representative lines for each construct are shown. While 11 out 12 of *2Rp:GUS* lines produced blue staining, none of the *R2p:GUS* lines gave a positive GUS signal. This experiment shows robust activty of the 2R promoter and confirmed RT-PCR data on the expression of the *At2g48160* gene (Figure 1). (C) To further analyze whether the 2R promoter can drive expression in the antisense orientation, the presence of spliced GUS transcripts was examined by RT-PCR. The expected 133 bp long product was readily detected in all analyzed *R2p:GUS* lines. Interestingly, a weak but specific product was also amplified in four out of six *2Rp:GUS* lines. These data are consistent with the RT-PCR analysis of TERRA at 2R (Figure 3D) and demonstrate that 2Rp promoter can initiate transcription into telomere.
(3.40 MB TIF)

Figure S3 Determination of the size of the telomeric siRNA. (A) Synthetic telomeric DNA and RNA oligonucleotides were used as size markers. DNA oligonucleotides are written in black. The telomeric G-RNAs (23 nt, 25 nt) and 23 nt C-RNA (written in blue) were synthesized by *in vitro* transcription from dsDNA produced by annealing the DNA oligonucleotides as indicated. (B) To determine the difference in migration between complementary telomeric RNA oligonucleotides, *in vitro* transcribed telomeric RNA as well as the indicated synthetic DNA oligonucleotides were separated by PAGE, electro-blotted onto a nylon membrane and sequentially hybridized with the CCCTAAA and TTTAGGG probes (only the right part of the membrane is shown after TTTAGGG hybridization). This experiment shows that 23 and 25 nt G-RNAs migrate like 25 and 27 nt TelG DNA oligonucleotides, respectively, while the 23 nt C-RNA migrates like the 22 nt TelG DNA oligonucleotide. This experiment demonstrates that C-RNA migrates faster than G-RNA of the corresponding size. (C) DNA oligonucleotides were used as size markers and separated together with plant siRNAs by PAGE, blotted onto a membrane and hybridized with the radioactively-labeled CCCTAAA probe. Migration of plant floral G-siRNAs (marked by asterisks) corresponds to the migration of 25–26 nt TelG DNA oligonucleotides. The signal was stripped after exposure and the membrane was rehybridized with the TTTAGGG probe for detection of the C-siRNAs (marked by asterisks). Because the signal from TelG DNA oligonucleotides was not completely stripped, we could use it as a marker to determine that plant C-siRNAs migrate like 24–25 nt TelG DNA oligonucleotides. Taking into account the difference in the migration of telomeric DNA and RNA (Figure S3B), we calculate that the size of plant G-siRNAs is 23–24 nt, and the size of the C-siRNAs is 24–25 nt.
(10.61 MB TIF)

Figure S4 Cytosine methylation in the subtelomeric regions 2R', 1L-2', and 1L-3'. Wild-type bisulfite-treated genomic DNA was used as a template for PCR with primers spanning subtelomeric regions 2R', 1L-2', and 1L-3' (Figure 2B). The diagrams representing distribution of 5-methylcytosines in individual clones were generated using CyMATE software for analysis of sequencing data of bisulfite-converted samples [50]. The data show almost a complete lack of DNA methylation in these regions.
(9.05 MB TIF)

Figure S5 Telomere length analysis in RdDM-deficient mutants. Southern analysis of Tru9I-digested genomic DNA hybridized with a telomeric probe. Each line represents a DNA sample extracted from a single plant. The telomere length in all analyzed mutants falls in the range typical for wild-type plants (2–5 kb).
(1.63 MB TIF)

Figure S6 Analysis of intrachromatid recombination in RdDM-deficient mutants. Intrachromatid telomere recombination leads to excision of telomeric extrachromosomal circular DNA molecules (t-circles). We used the highly sensitive t-circle amplification assay to analyze the level of t-circles in RdDM mutants [56]. Genomic DNA was digested with the AluI restriction enzyme and digestion-resistant t-circles were used as templates for primer extension via rolling circle amplification by the highly processive Phi29 polymerase. The high molecular weight products of rolling circle replication (indicated by an arrow) were separated from the bulk of digested genomic DNA by alkaline electrophoresis and were detected by Southern hybridization with a telomeric probe. Whereas a strong t-circle signal was obtained in *ku70* mutants that exhibit increased telomeric recombination [56], no t-circles, indicating an elevated level of recombination, were detected in the RdDM-deficient plants.
(1.30 MB TIF)

Table S1 Argonaute-associated telomeric siRNAs. The region of homology to the *Arabidopsis* telomeric sequence is indicated in red.
(0.03 MB XLS)

Table S2 Argonaute-associated siRNAs that uniquely align to subtelomeric regions.
(0.05 MB XLS)

Table S3 Primers used in this study.
(0.09 MB DOC)
