# Supplementary figures and images for: siRNA–Mediated Methylation of Arabidopsis Telomeres
Source: PLoS Genet. 2010 Jun 10;6(6):e1000986. doi: 10.1371/journal.pgen.1000986 (PMC2883606; doi:10.1371/journal.pgen.1000986)

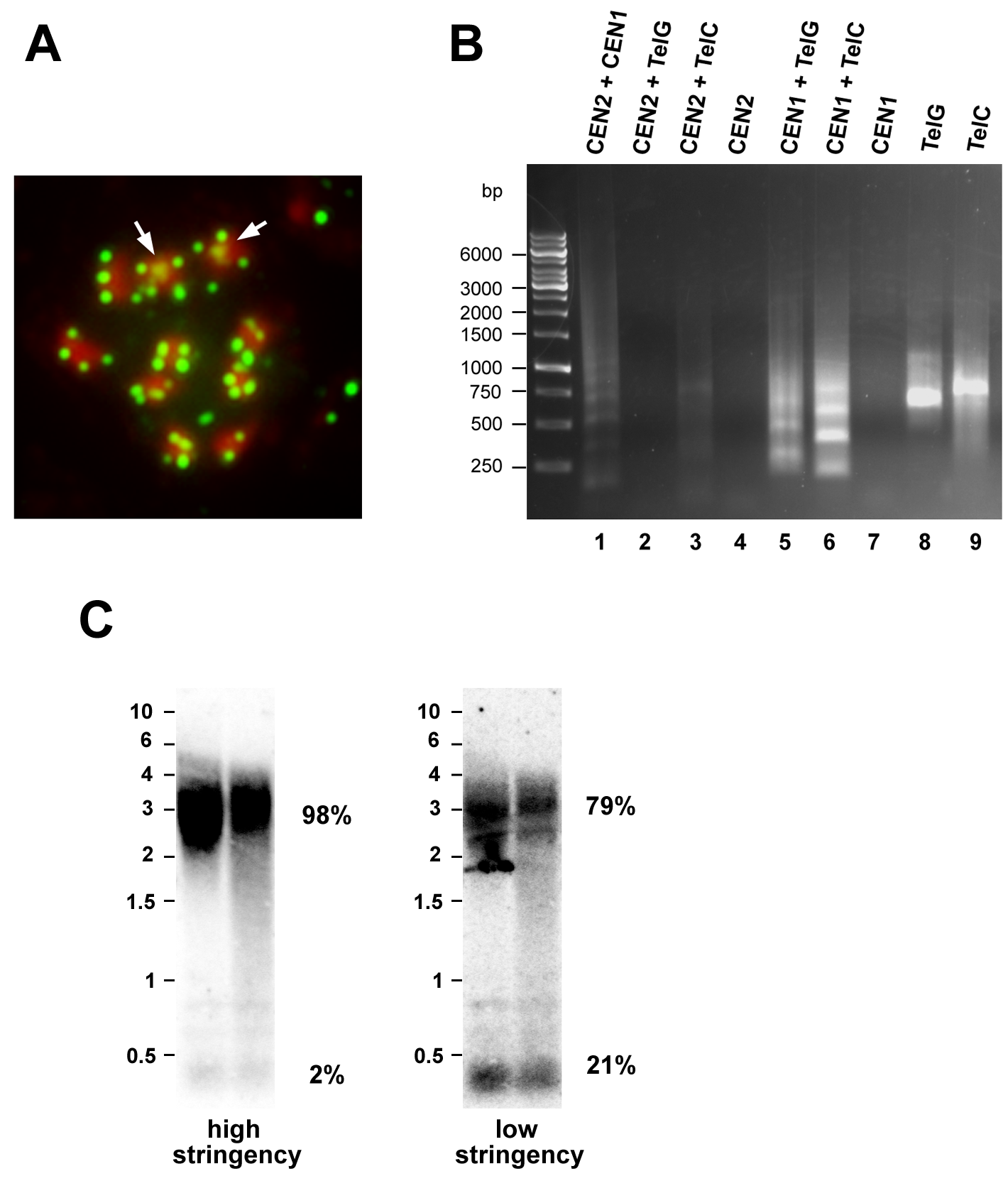

Supplement: Figure S1 — Localization of telomeric DNA in Arabidopsis centromeres. A survey of the Arabidopsis genome led to the identification of several regions carrying short stretches of telomeric sequence in the proximity of centromeres (Uchida et al., 2002; Vannier et al., 2009). Fluorescent in situ hybridization (FISH) on pachytene chromosomes further showed co-localization of CEN180 and telomeric signals at the centromere of chromosome 1 (Armstrong et al., 2002) (A) This centromere-localized telomeric DNA is also readily detectable by FISH on mitotic metaphase chromosomes. The picture shows a diploid metaphase figure with ten Arabidopsis chromosomes counterstained with DAPI (red). Green signals represent telomeric DNA; the chromosome pair carrying centromere-localized telomeric DNA is indicated by arrows. (B) The poor annotation of Arabidopsis centromeres precluded in silico identification of genomic loci carrying telomeric and CEN180 sequences in close proximity. To look for the presence of such loci in the Arabidopsis genome, we performed PCR with combinations of primers that flank the centromeric CEN180 satellite repeat (CEN1, CEN2) as well as with primers that anneal either to the C-rich or G-rich telomeric strands (TelC, TelG). The reaction with both centromeric primers resulted in a ladder whose periodicity corresponds to the size of the CEN180 repeat unit (180 bp; lane 1). Importantly, products were also amplified in reactions in which the CEN1 primer was combined with either of the telomeric primers (lanes 5 and 6), demonstrating that telomeric sequences are adjacent to the CEN180 repeat. Furthermore, strong amplification products were obtained in reactions containing a single telomeric primer (lanes 8 and 9), indicating the existence of sequences that contain telomeric repeats in inverted orientation. (C) Intrachromosomal telomeric sequences do not efficiently hybridize to a telomeric probe under the high stringency conditions. Genomic DNA was digested with TruI1 restrictio [file pgen.1000986.s001.tif]

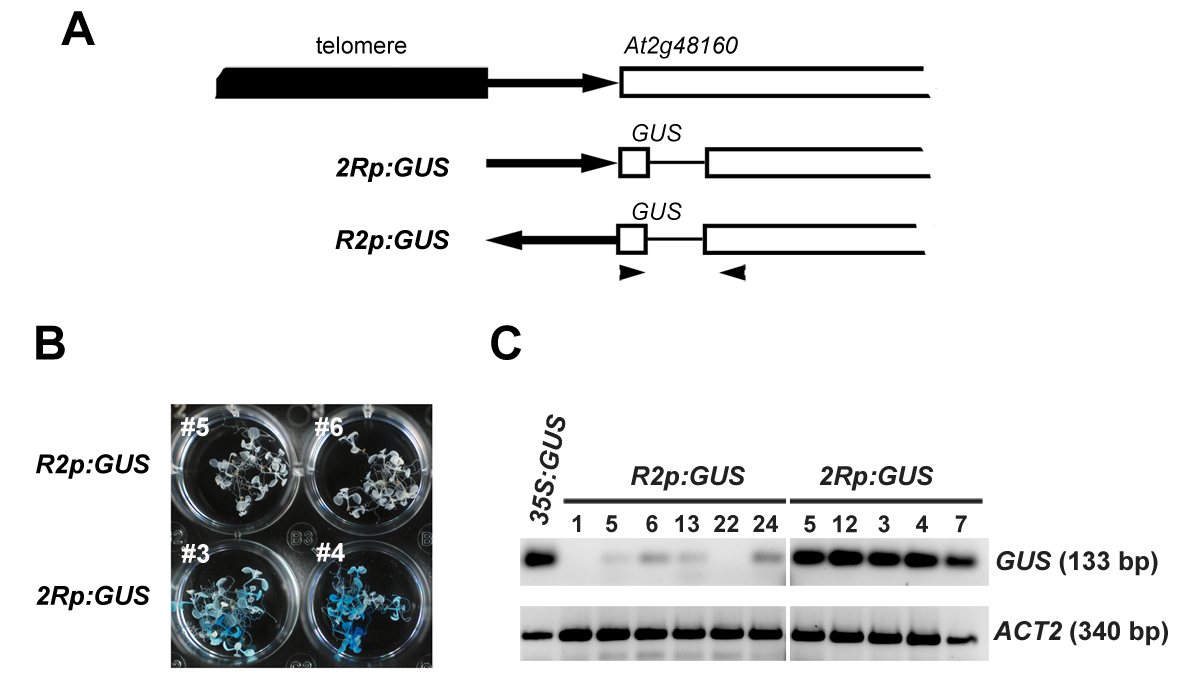

Supplement: Figure S2 — Analysis of bidirectional transcription of the GUS reporter gene from the 2Rp promoter. (A) Schematic representation of constructs used for this experiment. A ∼500 bp genomic fragment (indicated by arrow) localized between the telomere and At2g48160 was cloned in sense and antisense orientations in front of the GUS reporter gene containing a ∼200 nt intron (the intron is indicated by thin line; empty boxes represent exons). Resulting constructs (2Rp:GUS and R2p:GUS, respectively) were randomly inserted in Arabidopsis genome using Agorbacterium mediated transformation. T2 transgenic plants were analyzed for GUS expression by histochemical GUS assay and RT-PCR. Primers used for RT-PCR are indicated by arrows. (B) In total, T2 seedlings of 12 independent transgenic lines carrying the 2Rp:GUS construct, and 11 lines with the R2p:GUS were analyzed by histochemical GUS assay for the presence of GUS enzymatic activity. Two representative lines for each construct are shown. While 11 out 12 of 2Rp:GUS lines produced blue staining, none of the R2p:GUS lines gave a positive GUS signal. This experiment shows robust activty of the 2R promoter and confirmed RT-PCR data on the expression of the At2g48160 gene (Figure 1). (C) To further analyze whether the 2R promoter can drive expression in the antisense orientation, the presence of spliced GUS transcripts was examined by RT-PCR. The expected 133 bp long product was readily detected in all analyzed R2p:GUS lines. Interestingly, a weak but specific product was also amplified in four out of six 2Rp:GUS lines. These data are consistent with the RT-PCR analysis of TERRA at 2R (Figure 3D) and demonstrate that 2Rp promoter can initiate transcription into telomere. (3.40 MB TIF) [file pgen.1000986.s002.tif]

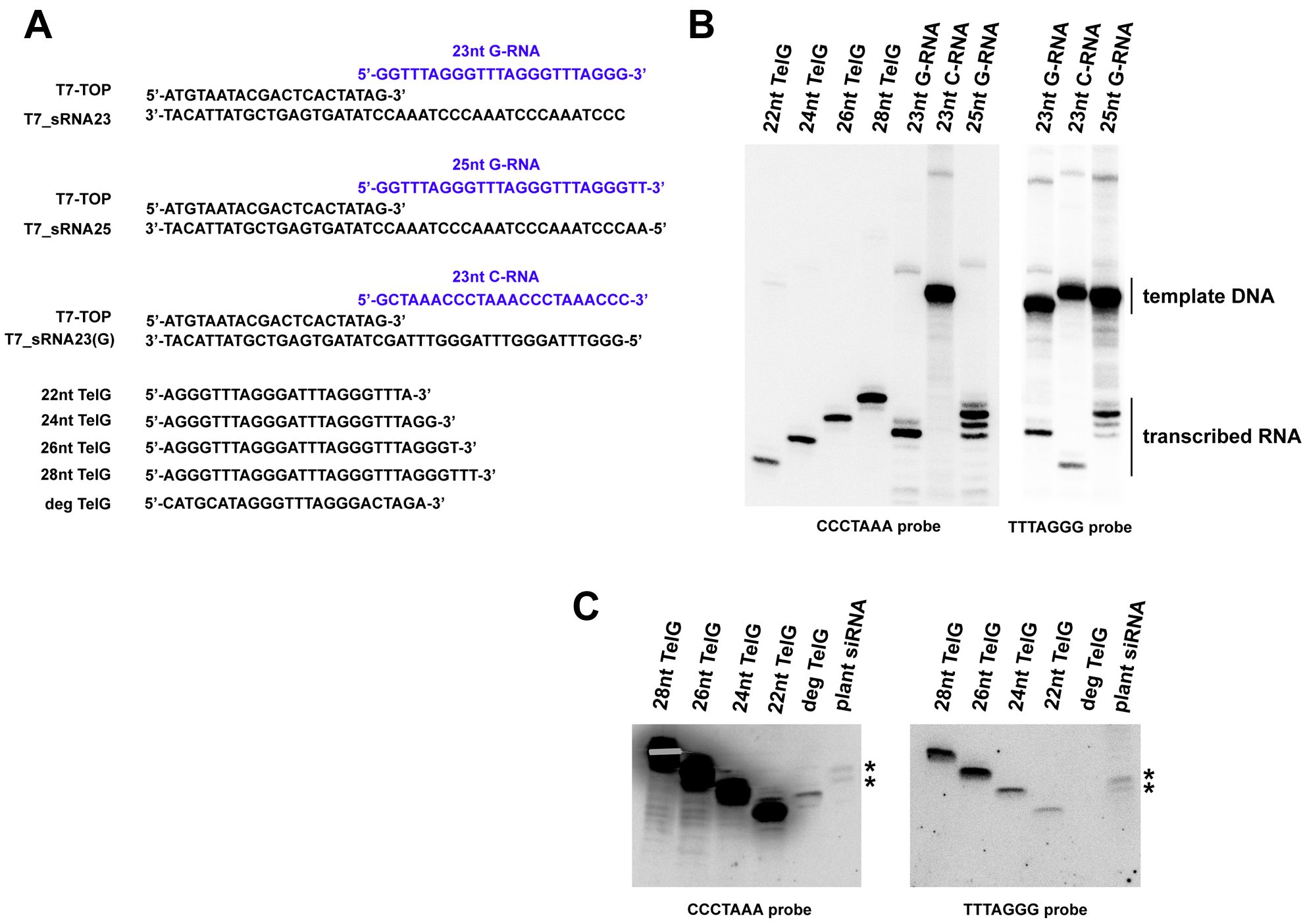

Supplement: Figure S3 — Determination of the size of the telomeric siRNA. (A) Synthetic telomeric DNA and RNA oligonucleotides were used as size markers. DNA oligonucleotides are written in black. The telomeric G-RNAs (23 nt, 25 nt) and 23 nt C-RNA (written in blue) were synthesized by in vitro transcription from dsDNA produced by annealing the DNA oligonucleotides as indicated. (B) To determine the difference in migration between complementary telomeric RNA oligonucleotides, in vitro transcribed telomeric RNA as well as the indicated synthetic DNA oligonucleotides were separated by PAGE, electro-blotted onto a nylon membrane and sequentially hybridized with the CCCTAAA and TTTAGGG probes (only the right part of the membrane is shown after TTTAGGG hybridization). This experiment shows that 23 and 25 nt G-RNAs migrate like 25 and 27 nt TelG DNA oligonucleotides, respectively, while the 23 nt C-RNA migrates like the 22 nt TelG DNA oligonucleotide. This experiment demonstrates that C-RNA migrates faster than G-RNA of the corresponding size. (C) DNA oligonucleotides were used as size markers and separated together with plant siRNAs by PAGE, blotted onto a membrane and hybridized with the radioactively-labeled CCCTAAA probe. Migration of plant floral G-siRNAs (marked by asterisks) corresponds to the migration of 25–26 nt TelG DNA oligonucleotides. The signal was stripped after exposure and the membrane was rehybridized with the TTTAGGG probe for detection of the C-siRNAs (marked by asterisks). Because the signal from TelG DNA oligonucleotides was not completely stripped, we could use it as a marker to determine that plant C-siRNAs migrate like 24–25 nt TelG DNA oligonucleotides. Taking into account the difference in the migration of telomeric DNA and RNA (Figure S3B), we calculate that the size of plant G-siRNAs is 23–24 nt, and the size of the C-siRNAs is 24–25 nt. (10.61 MB TIF) [file pgen.1000986.s003.tif]

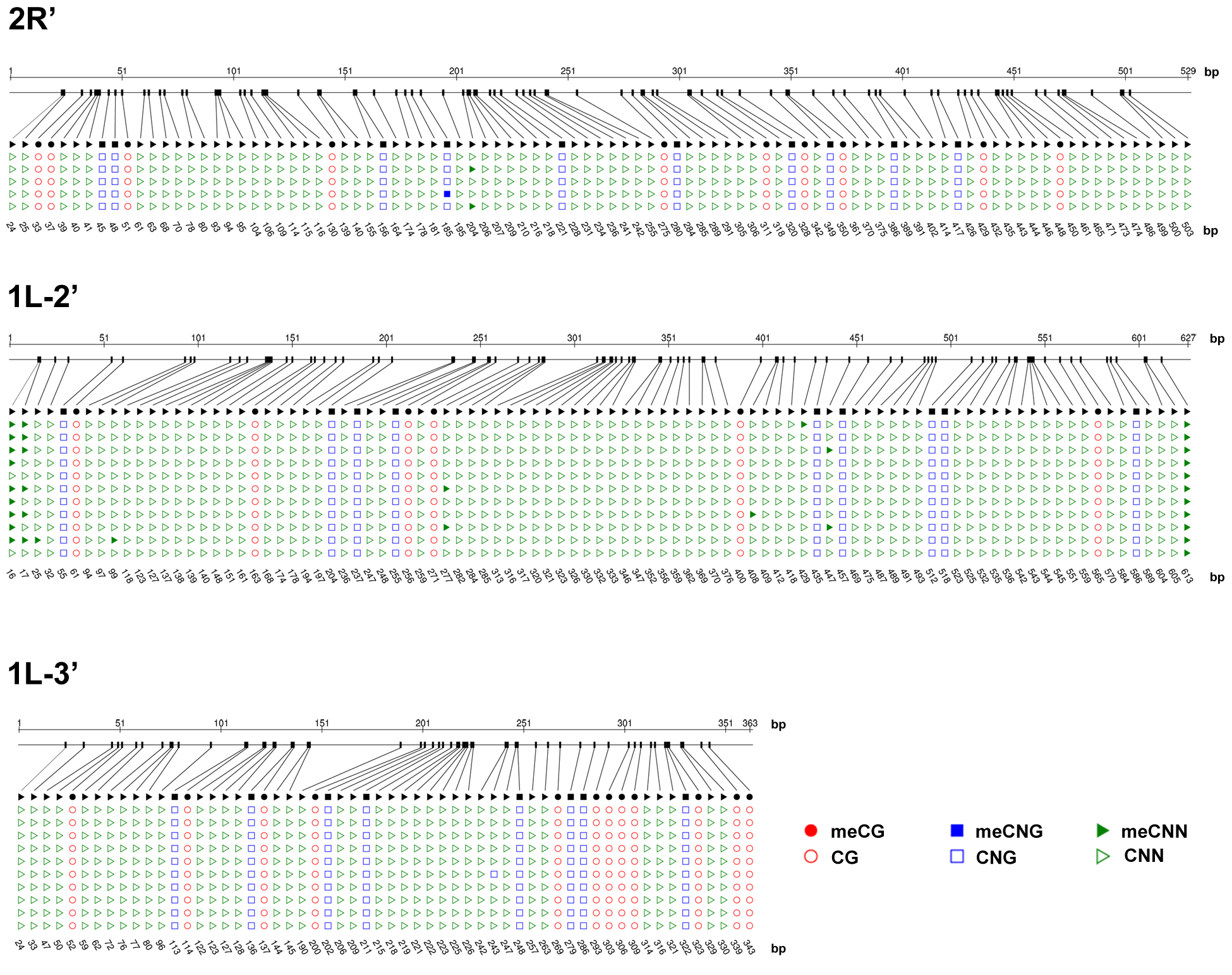

Supplement: Figure S4 — Cytosine methylation in the subtelomeric regions 2R', 1L-2', and 1L-3'. Wild-type bisulfite-treated genomic DNA was used as a template for PCR with primers spanning subtelomeric regions 2R', 1L-2', and 1L-3' (Figure 2B). The diagrams representing distribution of 5-methylcytosines in individual clones were generated using CyMATE software for analysis of sequencing data of bisulfite-converted samples [50]. The data show almost a complete lack of DNA methylation in these regions. (9.05 MB TIF) [file pgen.1000986.s004.tif]

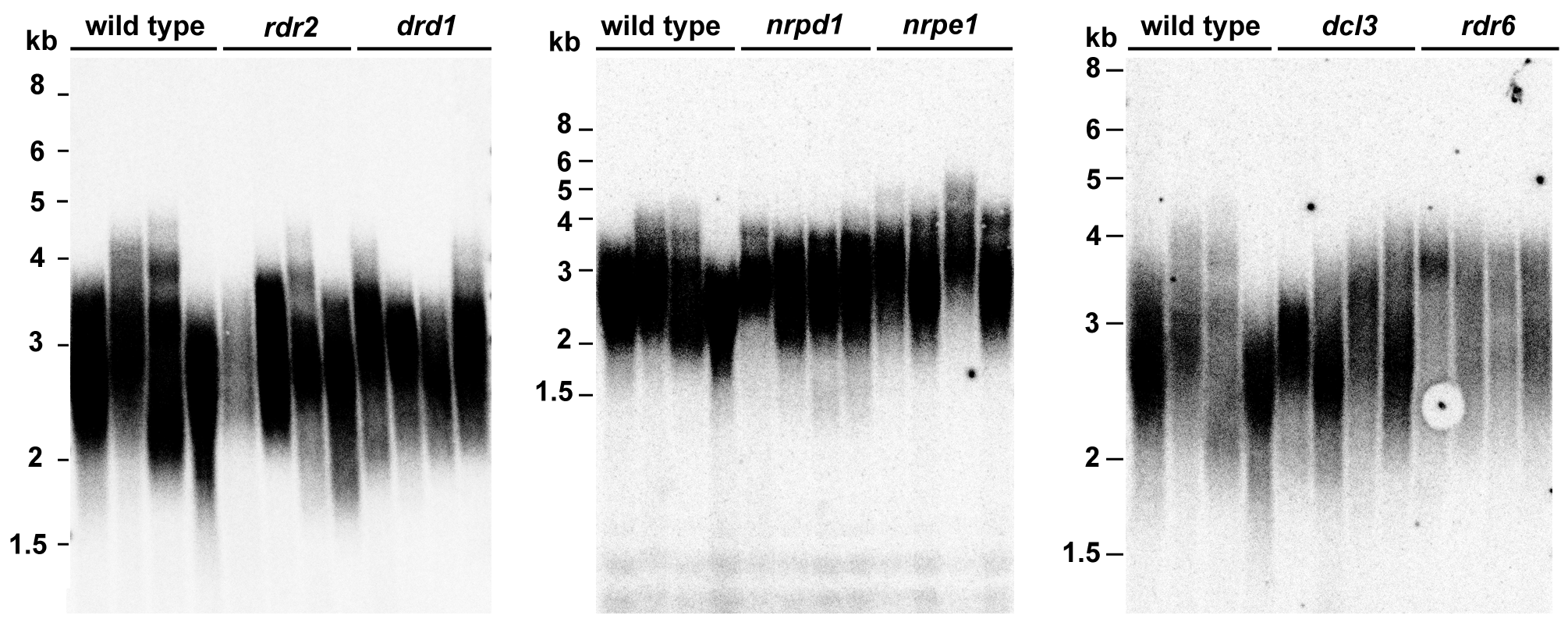

Supplement: Figure S5 — Telomere length analysis in RdDM-deficient mutants. Southern analysis of Tru9I-digested genomic DNA hybridized with a telomeric probe. Each line represents a DNA sample extracted from a single plant. The telomere length in all analyzed mutants falls in the range typical for wild-type plants (2–5 kb). (1.63 MB TIF) [file pgen.1000986.s005.tif]

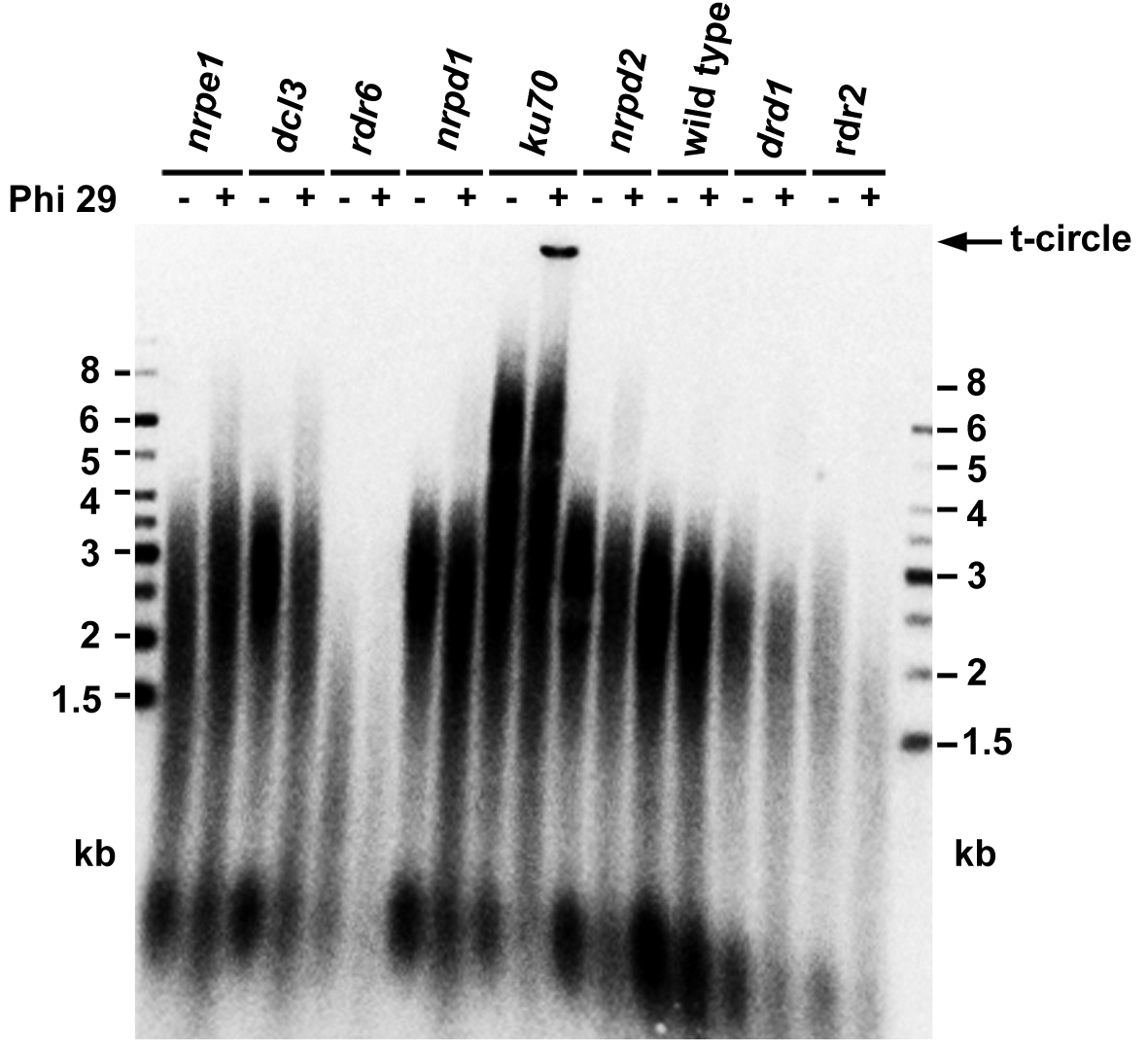

Supplement: Figure S6 — Analysis of intrachromatid recombination in RdDM-deficient mutants. Intrachromatid telomere recombination leads to excision of telomeric extrachromosomal circular DNA molecules (t-circles). We used the highly sensitive t-circle amplification assay to analyze the level of t-circles in RdDM mutants [56]. Genomic DNA was digested with the AluI restriction enzyme and digestion-resistant t-circles were used as templates for primer extension via rolling circle amplification by the highly processive Phi29 polymerase. The high molecular weight products of rolling circle replication (indicated by an arrow) were separated from the bulk of digested genomic DNA by alkaline electrophoresis and were detected by Southern hybridization with a telomeric probe. Whereas a strong t-circle signal was obtained in ku70 mutants that exhibit increased telomeric recombination [56], no t-circles, indicating an elevated level of recombination, were detected in the RdDM-deficient plants. (1.30 MB TIF) [file pgen.1000986.s006.tif]
