# Supplementary material for: siRNA–Mediated Methylation of Arabidopsis Telomeres
Source: PLoS Genet. 2010 Jun 10;6(6):e1000986. doi: 10.1371/journal.pgen.1000986 (PMC2883606; doi:10.1371/journal.pgen.1000986)
Supplement: Table S3 — Primers used in this study. (0.09 MB DOC) [file pgen.1000986.s009.doc]

**Table S3.**

| Name | Sequence | Purpose |
| --- | --- | --- |
| BS_R.bt1 | ATTTTTYYATAGGATAGATAGGG | Bisulfite sequencing of the 1L-0’ region |
| BS_F.bt2 | TCCAAACATAAACARTCCAAACAA | Bisulfite sequencing of the 1L-0’ region |
| metEND.BS_F | AAACATTTTACTATGAATACAT | Bisulfite sequencing of the 1L-2’ region |
| metEND.BS_R | GTTGAATTAGAGGAACCTTTGAT | Bisulfite sequencing of the 1L-2’ region |
| BS.BT.SUBG-R | AGTAAAAAATAATATGATTAAGA | Bisulfite sequencing of the 1L-3’ region |
| BS.BT.SUBG-F | ATCCTCCATTTTCACTAAACC | Bisulfite sequencing of the 1L-3’ region |
| 2R_Bis_R | TGATGATATTATTGTTGAATGGT | Bisulfite sequencing of the 2R’ region |
| 2R_Bis_F | ACATTTCCCATTAATAAACC | Bisulfite sequencing of the 2R’ region |
| 1LU | CAGTGGGGATTTTGTAGTG | RT-PCR analysis of *At1g01010* expression |
| 1LL | AAGGTGCCAAGTAAGGAAC | RT-PCR analysis of *At1g01010* expression |
| 1RU | TTCCCAGTCTCTGTCTCATC | RT-PCR analysis of *At1g80980* expression |
| 1RL | TGAACCACCCAAGAAGAAC | RT-PCR analysis of *At1g80980* expression |
| 1RU-2 | TGATAACGTATTGGTGTCTTC | RT-PCR analysis of *At1g80990* expression |
| 1RL-2 | ATAGACTTCATCACCGGAGCT | RT-PCR analysis of *At1g809910* expression |
| 2RU | CTGCCACTACTGTTGTTCTG | RT-PCR analysis of *At2g48160* expression |
| 2RL | CAATGCCTCTGAGATATGC | RT-PCR analysis of *At2g48160* expression |
| 3LU | CTTGAGCTATGGGGATTCTC | RT-PCR analysis of *At3g01015* expression |
| 3LL | CATGCCTCAGTTTCTTCATC | RT-PCR analysis of *At3g01015* expression |
| 4RU | CCCGAATTGGTTTCTGAATC | RT-PCR analysis of *At4g40100* expression |
| 4RL | TGCTCCTCTGGAACAGTTTC | RT-PCR analysis of *At4g40100* expression |
| 5LU | TGACCAGGGTTTTGTAGAC | RT-PCR analysis of *At5g01010* expression |
| 5LL | AGCAGATGGAGGAGATACTG | RT-PCR analysis of *At5g01010* expression |
| 5RU | TCTTCTCTTCTTGACTTTTC | RT-PCR analysis of *At5g67640* expression |
| 5R-BA | TGGAGGTTGTTTACACTTTGG | RT-PCR analysis of *At5g67640* expression |
| ACT2-1 | CTGCCGCTGTTGTTTCTCCT | RT-PCR analysis of *ACT2* (*At5g09810)* expression |
| ACT2-2 | CGTTGTAGAAAGTGTGATGCCA | RT-PCR analysis of *ACT2* (*At5g09810)* expression |
| (C3TA3)3 | (CCCTAAA)_3_ | RT of TERRA |
| M1bR | AAGAGACTCACATTCCCAAAT | RT for ARRET at 1L |
| 1R-10 | GGTAGTAATACTTATATCTTCATCATC | RT for ARRET at 1R & 4R |
| 2R-1 | GGCAAGTGTTGCAAGGC | RT for ARRET at 2R |
| 3L-7 | GGCCAAAATCAACGCTATCAAAC | RT for ARRET at 3L |
| 5L-11 | GTAAAGCCGCAAAACATAATTATAG | RT for ARRET at 5L |
| 5R-2 | CTATCATTTCTTATATCTCTCTC | RT for ARRET at 5R |
| 1R-8 | CAAGACATGGTAACTAATTAAGAATAAGAATC | RT-PCR for 1R & 4R TERRA/ARRET |
| 1R-9 | CATGTAGTAATCCCATTACATCAGCTT | RT-PCR for 1R & 4R TERRA/ARRET |
| Test1 | GATGAGCTTTGGCGGGACTG | RT-PCR for 2L/4L TERRA |
| Test2 | CCCTAAACCCTAGTGTTCG | RT-PCR for 2L/4L TERRA |
| 2R-2 | CTCCTAAAATATTTGCTAAGTAAATTGTCCAAC | RT-PCR for 2R TERRA/ARRET |
| Xba_2R Turn | TCTAGACTCATACAAAATTTTATGGTTA | RT-PCR for 2R TERRA/ARRET |
| 3L-5 | CCCTAATCTTTAGTTCCTAGACCCTAAATC | RT-PCR for 3L TERRA/ARRET |
| 3L-6 | CAACACAGAGAAGAAACAAGAGAAAG | RT-PCR for 3L TERRA/ARRET |
| 5L-2 | TTGCATAAAGCGTCACGTATAA | RT-PCR for 5L TERRA/ARRET |
| 5L-10 | CTGCCGCAAGCATGGGCTTG | RT-PCR for 5L TERRA/ARRET |
| 5R-1 | CAGGACGTGTGAAACAGAAACTACA | RT-PCR for 5R TERRA/ARRET |
| TAS5R-R1 | CGCTCTCATTGCGAGTGGTA | RT-PCR for 5R TERRA/ARRET |
| CEN1 | ATCAAGTCATATTCGACTCCA | PCR amplification of the CEN180 satellite repeat |
| CEN2 | CTCATGTGTATGATTGAGAT | PCR amplification of the CEN180 satellite repeat |
| TelG | (TTTAGGG)_4_ | PCR amplification of intrachromosomal telomeric DNA |
| TelC | (TAAACCC)_4_ | PCR amplification of intrachromosomal telomeric DNA |
| H3 F | CTCGATGTCGTATTCGCTGA | ChIP-PCR analysis of heterochromatin |
| H3 R | GCAACCTATCAACGCTTCGT | ChIP-PCR analysis of heterochromatin |
| B8 F | GCCACGAAAACCAAACAGAC | ChIP-PCR analysis of euchromatin |
| B8 R | CCGGAATTTCGATCAATCCT | ChIP-PCR analysis of euchromatin |
| Pat51-5 | CAACATGGCCCATTTAAGATTGAACG | ChIP-PCR analysis of 2R |
| 2R_Bis_F | ACATTTCCCATTAATAAACC | ChIP-PCR analysis of 2R |
| subG_F-chip | ACAGAGGATGATATTCAATCCA | ChIP-PCR analysis of 1L-3 |
| subG_R-chip | ATGGATCTCCGGTTTAATTGGT | ChIP-PCR analysis of 1L-3 |
| metEND_F-chip | ATGAGATGTTGAATTAGAGGAAC | ChIP-PCR analysis of 1L-2 |
| metEND-1_R-chip | ATCTTCCATATACAGTAATTAGA | ChIP-PCR analysis of 1L-2 |
| M3_F-chip | ATGCTCTATTTCTATCCTTGTA | ChIP-PCR analysis of 1L-1 |
| M4_R-chip | ATGTGTAAACGTGTGTCGATCA | ChIP-PCR analysis of 1L-1 |
| M0 | AGCGCTGTGGGATCAATCAT | ChIP-PCR analysis of 1L-0 |
| M1bR | AAGAGACTCACATTCCCAAAT | ChIP-PCR analysis of 1L-0 |
| M1 | AGATAGGGCCGACAAGATC | ChIP-qPCR analysis of 1L-0, RT-PCR for 1L TERRA/ARRET |
| M2 | TCAACCAGAGAAACCGGTTT | ChIP-qPCR analysis of 1L-0; RT-PCR for 1L TERRA/ARRET |
